# From Self-Esteem and Academic Performance to Anxiety: A Cross-Lagged Study of Chinese First-Generation College Students

**DOI:** 10.3390/bs16060999

**Published:** 2026-06-15

**Authors:** Xinqiao Liu, Ao Shen, Huirui Zhang

**Affiliations:** 1 School of Education, Tianjin University, Tianjin 300350, China; 2 Faculty of Education, The Open University of China, Beijing 100039, China

**Keywords:** first-generation college students, anxiety, self-esteem, academic performance, mental health

## Abstract

As the first generation in their families to pursue higher education, the mental health of first-generation college students has attracted significant attention from the academic community. Self-esteem and academic performance are significant factors influencing anxiety and mental health among first-generation college students. However, longitudinal research evidence specific to this group in China remains scarce. This study utilized two waves data, selecting a sample of 1024 first-generation college students (mean age 21.73; 55.18% male). Through follow-up surveys conducted at one-year intervals, a cross-lagged model was employed to systematically examine the longitudinal predictive relationships among self-esteem, academic performance, and anxiety. The results indicate significant negative correlations among self-esteem, academic performance, and anxiety. Cross-lagged analysis further indicated that self-esteem at T1 (β = −0.098, *p* < 0.05) and academic performance at T1 (β = −0.067, *p* < 0.05) were prospectively associated with lower anxiety at T2. This study reveals the longitudinal predictive associations among self-esteem, academic performance, and anxiety among China’s first-generation college students, providing empirical evidence for universities to improve their mental health support systems by focusing on the self-esteem development of this group and offering targeted academic support.

## 1. Introduction

In recent years, China’s higher education sector has continued to expand, entering a phase of universal access recognized worldwide. By 2024, the gross enrollment rate reached 60.8%, and an increasing number of students have gained access to higher education. At the same time, the number of first-generation college students has grown significantly, accounting for more than 70% of the total college student population (H. Zhang et al., 2016). Globally, there are significant regional variations in the proportion of first-generation college students. According to a report released by the OECD, the average proportion of first-generation college students among OECD member countries is approximately 46%, with Japan having the lowest proportion at just 24% and Italy the highest at 72% (OECD, 2014). According to data from the Eurostudent IV 2008–2011 report, the average proportion of first-generation college students across European countries was approximately 50%, with Denmark having the lowest proportion at 21% and Portugal the highest at 76% (Orr et al., 2011). By 2024, the average proportion of first-generation college students in European countries had fallen to approximately 41%, with Denmark and Norway having the lowest proportion at 23% and Portugal still having the highest at 60% (Hauschildt et al., 2024). According to data from the National Center for Education Statistics (NCES), the proportion of first-generation college students in the United States declined from approximately 33.5% in the 2011–2012 academic year (Skomsvold, 2014) to approximately 23.9% in the 2015–2016 academic year (Campbell & Wescott, 2019). These data indicate that, although the number and proportion of first-generation college students vary across different countries and regions, they have become a significant and indispensable group within the global higher education system.

Anxiety is a common negative emotion characterized by tension, worry, and fear, often beginning in childhood, adolescence, and early adulthood (Craske & Stein, 2016). Numerous studies have shown that college students are at a higher risk of developing anxiety (Ozen et al., 2010; Wang et al., 2020; W. Li et al., 2022), which not only affects their mental health but also has a negative impact on their academic performance (J. Zhang et al., 2024). Existing research has confirmed that frequent smoking (Ramón-Arbués et al., 2020), poor-quality social support (Hefner & Eisenberg, 2009), adverse family economic circumstances (Eisenberg et al., 2007), and poor sleep quality (Ghrouz et al., 2019) are all significant contributors to anxiety among college students. Anxiety issues are particularly pronounced among first-generation college students. Compared to non-first-generation college students, they often face greater challenges (Martinez et al., 2009; Beattie, 2018) and are subjected to more stressors (Wilbur, 2021). They primarily come from disadvantaged backgrounds (Lohfink & Paulsen, 2005) and have lower family socioeconomic status (Jenkins et al., 2013). They face more severe financial difficulties and academic pressures (House et al., 2020), and they perceive significantly poorer faculty-student relationships, peer relationships, and campus support (X. Li et al., 2023). These challenges collectively form the practical foundation for the heightened susceptibility to anxiety among first-generation college students, making them a high-risk group for mental health that requires urgent attention (Gao et al., 2020; Noel et al., 2023).

To gain a deeper understanding of the mechanisms underlying anxiety among first-generation college students, self-esteem and academic performance are two factors that warrant particular attention. Self-esteem, defined as an individual’s subjective assessment of their self-worth (Donnellan et al., 2011), has been widely shown to moderate anxiety. Related studies indicate that low self-esteem is closely associated with anxiety (Zeigler-Hill, 2011), students with lower self-esteem are more prone to anxiety (Ratanasiripong et al., 2018), and high self-esteem can effectively improve mental health (Orth & Robins, 2022). Academic performance, as significant feedback of learning outcomes, is one of the primary drivers of emotional responses in educational settings and has a significant impact on various emotions, including anxiety (Goetz et al., 2018). Poor academic performance can have a negative impact on students’ mental health, thereby increasing the likelihood of anxiety (Stallman, 2010). For first-generation college students, these two factors hold particular significance. They often view higher education as the primary pathway to achieving upward social mobility (Tian & Zhang, 2025). Consequently, they place higher expectations and emotional investment in their academic performance, making academic setbacks more likely to trigger intense negative emotional reactions. At the same time, social comparison and the pressures of cultural adaptation also subject their self-esteem to more severe challenges.

The anxiety, self-esteem, and academic performance of first-generation college students have become a major focus of ongoing attention among educators worldwide. Constructing a cross-lagged model to examine the longitudinal predictive relationships among self-esteem, academic performance, and anxiety among first-generation college students—particularly those in the context of Chinese higher education—can help systematically reveal the dynamic interrelationships among these three factors and provide empirical evidence to guide universities in their mental health education initiatives.

## 2. Literature Review

### 2.1. Definition and Group Characteristics of First-Generation College Students

Definitions of first-generation college students vary slightly across countries and studies due to a lack of consensus (Toutkoushian et al., 2018), but core criteria consistently revolve around “parents’ higher education experience”. First-generation college students, in the narrow sense, refer to those whose parents not only lack a college degree but also have no higher education-related learning experience whatsoever (Ishitani, 2006). In the broad sense, first-generation college students include those whose parents may have received some college education—such as holding a community college certificate or an associate’s degree—but have not earned a formal university degree (Engle & Tinto, 2008). On the basis of practical needs and the characteristics of China’s education system, this study adopts a broad definition, identifying first-generation college students as those whose parents or guardians have never received higher education or who attended but did not complete university studies.

Compared with non-first-generation college students, the first-generation college students cohort has the following characteristics. First, first-generation college students often come from families in lower socioeconomic strata. Owing to their families’ lower socioeconomic status (Hunt et al., 2018; Norodien-Fataar, 2018) and lack of cultural capital (Kenny & Perez, 1996; Barsegyan & Maas, 2024), they struggle to access learning resources and cultural support commensurate with higher education (Sy et al., 2011). Consequently, they face significant life pressures (Pike et al., 2008) and are more inclined to pursue higher education to help their families improve their economic circumstances after graduation (Bui, 2002). Second, first-generation college students often struggle with academic adjustment and exhibit low academic engagement (Soria & Stebleton, 2012), frequently resulting in poorer academic performance (X. Li et al., 2023). This, in turn, leads to increased academic stress (House et al., 2020) and a significantly increased risk of dropping out (Cataldi et al., 2018). Furthermore, they demonstrate a lower propensity to pursue further education after graduation (Mullen et al., 2003; Carlton, 2015). Third, at the psychological level, first-generation college students face a wider range of stressors (Wilbur, 2021). They often bear the heavy expectations associated with their family’s social mobility (Spiegler & Bednarek, 2013) and exhibit more severe anxiety (Jeong et al., 2023) and depressive symptoms (Rocha et al., 2025) while also being less likely to seek mental health treatment (Lipson et al., 2023). These characteristics make exploring the factors influencing the mental health and anxiety of first-generation college students a significant topic in educational research.

### 2.2. The Relationship Between Self-Esteem and Anxiety

Existing research consistently confirms a significant negative relationship between anxiety and self-esteem. For example, in a study of 61 German students, Riketta (2004) found a clear negative correlation between self-esteem and anxiety. Surveys of specific populations have further validated this conclusion. Pratama and Tjhin (2024) found that among a group of Indonesian high school students, most high school students experience anxiety, and those with low self-esteem are at a significantly higher risk of anxiety than those with high self-esteem. Among Palestinian undergraduate nursing students, there was a negative correlation between social anxiety and self-esteem, and lower social anxiety was significantly associated with increased self-esteem (Ayed et al., 2024). A study of 234 Chinese university students also found that self-esteem was significantly negatively correlated with both body image anxiety and social anxiety, and acted as a mediator in the process by which body image anxiety influences social anxiety (Liao et al., 2023). This negative correlation is consistent across cultural contexts. Studies by Kim and Koh (2018) involving 313 South Korean university students and Ramón-Arbués et al. (2020) involving 1074 Spanish university students both yielded similar results.

Related studies have also revealed the longitudinal predictive role of self-esteem on anxiety. A four-year longitudinal study of 2473 Chinese college students indicated that low self-esteem significantly predicts subsequent anxiety, and this effect gradually increases with academic year (Liu et al., 2022). A cross-lagged analysis by Cao & Liu (2024), based on two waves of longitudinal data, further confirmed that there is a significant negative correlation between self-esteem and anxiety, and that self-esteem significantly and negatively predicts anxiety. Furthermore, self-esteem is not only a significant predictor of test anxiety (Von der Embse et al., 2018) but also effectively buffers the negative impact of bullying victimization on social anxiety (Boulton & Macaulay, 2023). Furthermore, some studies have found that self-esteem and anxiety levels exhibit a bidirectional predictive relationship at the individual level, meaning that low self-esteem increases the risk of subsequent anxiety, while anxiety further lowers self-esteem levels (W. Li et al., 2023). Based on the above research background, this study proposes the following hypotheses.

**Hypothesis 1.**  *There is a significant negative correlation between self-esteem and anxiety among first-generation college students.*

**Hypothesis 2.**  *Self-esteem levels among first-generation college students significantly and negatively predict their anxiety levels.*

### 2.3. The Relationship Between Academic Performance and Anxiety

The relationship between academic performance and anxiety has long been a major focus of academic research. Seipp (1991) conducted a meta-analysis to synthesize previous research findings, revealing a significant negative correlation between academic performance and anxiety, and noting that the strength of this negative association may be influenced by the study population, measurement instruments, and contextual factors. Subsequent studies have also confirmed this relationship. A survey of 100 undergraduate students in Malaysia revealed that female students had slightly higher anxiety levels than male students, and that academic performance was significantly negatively correlated with anxiety levels (Kaswadi et al., 2018). A specific study of engineering students indicated a negative association between high levels of academic anxiety and lower academic performance (Vitasari et al., 2010). Research on organic chemistry courses in higher education has also found a weak but significant negative correlation between academic performance and anxiety (Gibbons et al., 2018). At the same time, some studies suggest that academic performance serves as a predictor of anxiety. A longitudinal study by Meece et al. (1990) of students in grades 7 through 9 found that students’ performance in mathematics could predict their subsequent levels of math anxiety. A large-scale longitudinal study conducted by Pekrun’s team further systematically validated this predictive relationship. Their five-year longitudinal study of 3425 German students in grades 5–9 demonstrated a negative association between academic performance in mathematics and subsequent academic anxiety (Pekrun et al., 2017). Subsequent research, through more refined within-subject analyses, further suggested that fluctuations in academic performance can predict changes in an individual’s anxiety levels (Pekrun et al., 2023). Furthermore, a longitudinal study by Brook and Willoughby (2015) of a college student population found that academic performance may negatively predict an individual’s level of social anxiety through the mediating effect of social connectedness. Based on previous research, we propose the following hypotheses.

**Hypothesis 3.**  *There is a significant negative correlation between academic performance and anxiety among first-generation college students.*

**Hypothesis 4.**  *Academic performance among first-generation college students significantly and negatively predicts their anxiety levels.*

### 2.4. The Present Study

Existing research has confirmed that both self-esteem and academic performance are significantly negatively correlated with anxiety; however, several research gaps remain. First, most studies employ cross-sectional designs, making it difficult to reveal longitudinal predictive relationships among variables. Second, while existing research has primarily focused on the general college student population, systematic studies targeting the specific group of first-generation college students remain relatively scarce. Given that first-generation college students constitute a significant proportion of China’s higher education population and face unique challenges, conducting specialized research on this group holds significant innovative and research value. Therefore, to address the aforementioned research limitations, this study adopts a large-scale longitudinal tracking design. By collecting survey data at two time points and employing a cross-lagged model, it systematically investigates the longitudinal predictive effects of self-esteem and academic performance on anxiety among first-generation college students. This approach effectively addresses the shortcomings of existing research regarding population specificity and research methods, providing empirical evidence for a deeper understanding of the pathways through which anxiety affects this group.

## 3. Method

### 3.1. Participants and Procedure

The data for this study were drawn from the Beijing College Students Panel Survey, a large-scale longitudinal study designed to systematically examine the academic and daily lives of college students during their time at university. The study employed a longitudinal design and recruited students from a total of 16 universities, including Peking University, Tsinghua University, and Renmin University of China, among others. Data were collected via self-administered questionnaires. The survey was conducted in two phases: Phase 1 (Time 1, abbreviated as T1) served as the baseline data collection, while Phase 2 (Time 2, abbreviated as T2) involved a follow-up survey of the same group of respondents one year later, thereby yielding two rounds of longitudinal data. All participants voluntarily enrolled in the study and signed informed consent forms. Multiple studies using this dataset have confirmed that the data are highly reliable (Liu & Wang, 2024; Liu et al., 2024a).

Regarding the selection of study participants, based on the definition of first-generation college students, students whose father or mother had an educational attainment of “college or higher” were excluded from the sample. This resulted in a valid sample of 1024 participants at T1, and 1021 participants remained as the valid sample at T2. Subsequently, a *t*-test was conducted to further analyze whether sample attrition was random. The results showed no significant differences between the attrition group and the retention group in terms of gender, age, self-esteem, academic performance, and anxiety (*p* > 0.05), confirming that participant attrition was random. Among the participants, there were 565 males (55.18%) and 459 females (44.82%); 917 participants were Han Chinese (89.73%). The mean age at T1 was approximately 21.73 years (SD = 0.907), and at T2 it was approximately 22.73 years (SD = 0.904), consistent with the expected age progression in a longitudinal study of the same sample.

### 3.2. Measures

#### 3.2.1. Self-Esteem

This study employed the self-esteem scale (SES) developed by American scholar Rosenberg (1965) to measure the self-esteem of first-generation college students. The SES comprises 10 items, including 5 positively worded statements and 5 negatively worded statements. Each item features five response options ranging from “1” (indicating “completely disagree”) to “5” (indicating “completely agree”). Owing to significant differences in the interpretation of the eighth item, “I wish I could have more respect for myself,” between Chinese and Western cultures, this item was excluded from the data analysis in this study to ensure measurement accuracy (Han et al., 2005). The participants’ total scores ranged from 9 to 45 points, with higher scores indicating stronger self-esteem. The calculations revealed Cronbach’s alpha coefficients of α = 0.878 for SES at T1 and α = 0.878 at T2, demonstrating excellent measurement reliability.

#### 3.2.2. Academic Performance

We collected data on students’ academic performance at two time points, T1 and T2, and assessed their academic performance levels based on their rankings within their classes. Given the significant variations in student enrollment, course difficulty, and grading standards across different majors in Chinese universities, relative rankings provide a more objective and reliable reflection of students’ academic performance across disciplines. To facilitate subsequent statistical analysis and longitudinal predictive modeling, and in reference to previously published literature (Liu et al., 2024b), this study categorizes students’ relative rankings into ten groups, specifically: top 10% of the class = rank 10, top 20% = rank 9, top 30% = rank 8, top 40% = rank 7, top 50% = rank 6, top 60% = rank 5, top 70% = rank 4, top 80% = rank 3, top 90% = rank 2, top 100% = rank 1. This means that a higher score indicates better academic performance.

#### 3.2.3. Anxiety

This study employed the Depression Anxiety and Stress Scale (DASS-42) to measure anxiety levels among first-generation college students (Lovibond & Lovibond, 1995). The DASS-42 is a 42-item questionnaire comprising three self-report scales designed to measure three negative emotional states: depression, anxiety, and stress. It is a clinically validated psychological assessment tool widely used in psychological research worldwide. The Anxiety Scale comprises 14 items scored on a 4-point scale ranging from “0” (indicating “Did not apply to me at all”) to “3” (indicating “Applied to me very much, or most of the time”). The participants’ total scores range from 0 to 42, with higher scores indicating higher levels of anxiety. The calculations revealed that this scale demonstrated high reliability in the present study: Cronbach’s alpha coefficient at T1 was α = 0.850, and at T2, it was α = 0.868.

### 3.3. Data Analysis

This study utilized Stata 15.0 and Mplus 7.4 for data analysis. First, Stata 15.0 was used to conduct descriptive statistics and correlation analyses of first-generation college students’ self-esteem, academic performance, and anxiety. Second, a cross-lagged model was constructed to empirically investigate the longitudinal predictive effects of self-esteem and academic performance on anxiety among first-generation college students. The cross-lagged model aims to examine the prospective predictive effects of one variable on another while controlling for the correlation between variables at the same time point and the cross-temporal stability of the variables themselves (Orth et al., 2021). The academic performance variable was treated as a continuous observed variable in the SEM. Following recommendations in the relevant literature, we divided the items measuring self-esteem and anxiety into three groups each to reduce measurement error (Hau & Marsh, 2004; Yang et al., 2010). Finally, we used Mplus 7.4 to test the model fit of the cross-lagged model and calculate the cross-lagged results.

**Figure 1:**
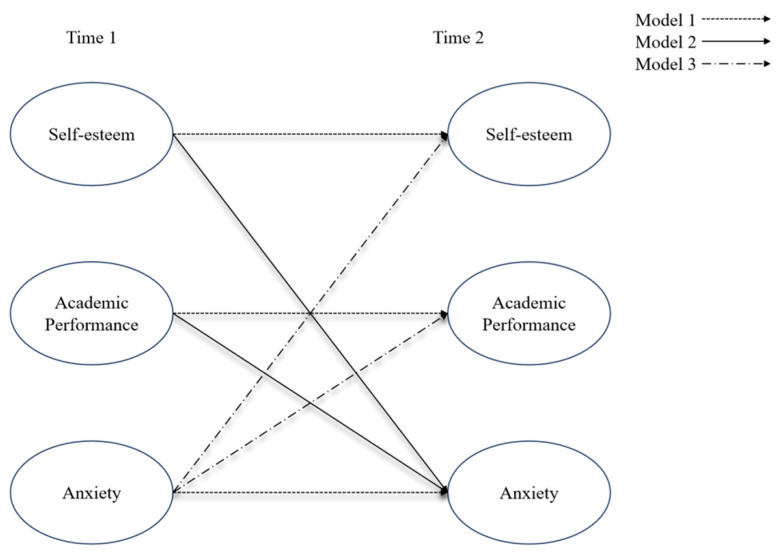
Cross-lagged Model of Self-Esteem, Academic Performance, and Anxiety. Note: Model 4 is a bidirectional model that incorporates all autoregressive paths and cross-lagged paths from Models 1, 2, and 3.

The cross-lagged model is shown in Figure 1. Model 1 is an autoregressive model used to examine the stability of self-esteem, academic performance, and anxiety at T1 and T2, respectively. Model 2 builds upon the autoregressive path (Model 1) by adding two cross-lagged paths from self-esteem and academic performance at T1 to anxiety at T2, to investigate the predictive relationships between self-esteem and academic performance, and anxiety. Model 3 builds upon the autoregressive path (Model 1) by adding two cross-lagged paths from anxiety at T1 to self-esteem and academic performance at T2, to investigate the predictive role of anxiety on self-esteem and academic performance. Model 4 is a bidirectional model that includes all previous autoregressive and cross-lagged paths, used to analyze the predictive relationships among self-esteem, academic performance, and anxiety.

In Mplus 7.4, we re-estimated structural equation models (SEMs) using robust maximum likelihood estimation (MLR). Missing data were handled using full-information maximum likelihood (FIML) under the assumption of missing at random (MAR). Since FIML directly uses all available data, no coding of missing values is required. For comparisons of nested models, we employed robust scaled chi-square difference tests.

Additionally, to assess the fit of each model, we employed metrics such as the root mean square error of approximation (RMSEA), standardized root mean square residual (SRMR), comparative fit index (CFI), and Tucker–Lewis index (TLI) (Bentler & Bonett, 1980; Hu & Bentler, 1998). Typically, a model is considered to fit well if RMSEA ≤ 0.08, SRMR ≤ 0.08, CFI ≥ 0.95, or TLI > 0.90. This study evaluates and compares the models based on the aforementioned criteria.

## 4. Results

### 4.1. Descriptive and Correlation Analysis

Table 1 shows the means, standard deviations, and correlation coefficients for self-esteem, academic performance, and anxiety at time points T1 and T2. Descriptive analysis results show that the self-esteem scores for first-generation college students were 36.377 (SD = 6.144) at T1 and 36.271 (SD = 5.912) at T2, indicating a slight decline in self-esteem levels. Academic performance scores were 6.334 (SD = 2.41) at T1 and 6.554 (SD = 2.357) at T2, indicating a slight improvement in academic performance. According to the DASS-42 user manual, anxiety levels are categorized based on total scores into five grades: Normal (0–7), Mild (8–9), Moderate (10–14), Severe (15–19), and Extremely Severe (20 and above). In this study, the anxiety score at T1 was 8.192 (SD = 5.999), falling within the Mild range, while the score at T2 was 7.828 (SD = 6.129), slightly above the upper limit of the Normal range. This indicates that the anxiety levels of first-generation college students were generally within the mild range but showed some alleviation.

The results of the correlation analysis indicate that, at each time point, self-esteem and academic performance were significantly negatively correlated with anxiety. Specifically, self-esteem at T1 was negatively correlated with anxiety at T1 (r = −0.412, *p* < 0.05), self-esteem at T2 was negatively correlated with anxiety at T2 (r = −0.436, *p* < 0.05), academic performance at T1 was negatively correlated with anxiety at T1 (r = −0.073, *p* < 0.05), and academic performance at T2 was negatively correlated with anxiety at T2 (r = −0.130, *p* < 0.05). Across time points, self-esteem at T1 was negatively correlated with anxiety at T2 (r = −0.327, *p* < 0.05), and academic performance at T1 was negatively correlated with anxiety at T2 (r = −0.129, *p* < 0.05). These findings support Hypotheses 1 and 3, indicating that the negative correlations among self-esteem, academic performance, and anxiety among first-generation college students are relatively stable, and they also lay an empirical foundation for future research.

### 4.2. Model Comparison

The fit indices of the models are presented in Table 2. Model 1 indicates that the fit indices for the autoregressive analysis of self-esteem, academic performance, and anxiety are good (RMSEA = 0.072, SRMR = 0.058, CFI = 0.954, TLI = 0.941), and the autoregressive effects are significant, suggesting that the measured variables exhibit good stability between T1 and T2. Model 2 tested the predictive role of self-esteem and academic performance at T1 on anxiety at T2. This model exhibited good fit (RMSEA = 0.071, SRMR = 0.047, CFI = 0.956, TLI = 0.942), with a significant difference compared to Model 1 (Δχ^2^ = 17.577, *p* < 0.05), indicating that Model 2 had a better fit than Model 1. Model 3 examined the predictive role of anxiety at T1 on self-esteem and academic performance at T2, showing good model fit (RMSEA = 0.073, SRMR = 0.054, CFI = 0.955, TLI = 0.939). Although Model 3 showed acceptable model fit, it did not fit significantly better than Model 1. Model 4 is used to examine the bidirectional relationships among self-esteem, academic performance, and anxiety, and exhibits good model fit (RMSEA = 0.072, SRMR = 0.045, CFI = 0.956, TLI = 0.940). Overall, Model 4 more comprehensively addresses the research objectives of this study, exhibits better model fit than Model 1 (Δχ^2^ = 20.354, *p* < 0.05), and further enhances the robustness and reliability of the research findings.

### 4.3. Cross-Lagged Analysis

The standardized stability coefficients and cross-lagged coefficients for Models 1–4 are shown in Table 3; all coefficients reported are standardized estimates. Overall, the autoregressive effects of all variables from T1 to T2 were significant (*p* < 0.05), indicating that all variables exhibited good temporal stability. In Model 4, which served as the primary analytical framework, autoregressive path analysis revealed that the autoregressive coefficient for self-esteem was β = 0.551, *p* < 0.05; the autoregressive coefficient for academic performance was β = 0.828, *p* < 0.05; and the autoregressive coefficient for anxiety was β = 0.532, *p* < 0.05. Cross-lagged path analysis revealed that the cross-lagged coefficient for the path from self-esteem at T1 to anxiety at T2 was β = −0.098, *p* < 0.05, and the cross-lagged coefficient from academic performance at T1 to anxiety at T2 was β = −0.067, *p* < 0.05, indicating that self-esteem and academic performance at T1 could significantly and negatively predict anxiety at T2, respectively. Furthermore, the cross-lagged coefficient β for anxiety at T1 to self-esteem at T2 was −0.058, *p* > 0.05, and the cross-lagged coefficient from anxiety at T1 to academic performance at T2 was β = −0.003, *p* > 0.05, indicating that anxiety at T1 did not significantly predict self-esteem or academic performance at T2. Hypotheses 2 and 4 were supported. Both self-esteem and academic performance were found to be significant predictors of anxiety, indicating that first-generation college students with higher self-esteem and better academic performance tend to have lower anxiety levels.

## 5. Discussion

This study focuses on China’s first-generation college students and employs a cross-lagged model to examine the longitudinal relationships among self-esteem, academic performance, and anxiety. It contributes to a deeper understanding of the longitudinal predictors of anxiety among first-generation college students and provides timely and forward-looking empirical evidence to help universities address this group’s self-esteem development and academic adjustment, as well as optimize mental health support strategies.

The results of the descriptive analysis indicate that, compared to T1, first-generation college students’ self-esteem levels declined slightly at T2, while their academic performance improved slightly, consistent with findings from previous studies (Gao et al., 2022). Furthermore, although anxiety levels decreased somewhat at T2, they remained within the mild range. This indicates that anxiety is prevalent among first-generation college students, consistent with previous research (Ozen et al., 2010; Wang et al., 2020; W. Li et al., 2022). Compared to non-first-generation college students, first-generation college students often face greater challenges in academic adjustment and higher levels of psychological stress (Wilbur, 2021). They may be at a disadvantage in terms of financial support from their families, access to academic resources, and the accumulation of social capital (Sy et al., 2011; Hunt et al., 2018), making them more likely to experience higher levels of anxiety during their college years (Jeong et al., 2023). The results of the correlation analysis indicate that, for first-generation college students, there is a significant negative correlation between self-esteem, academic performance, and anxiety, whether measured at the same time or at different times, further supporting the findings of previous studies (Seipp, 1991; Riketta, 2004; Vitasari et al., 2010; Ayed et al., 2024). This suggests that higher levels of self-esteem and good academic performance are often accompanied by lower levels of anxiety.

The results of the cross-lagged model analysis indicate that, first, self-esteem levels among first-generation college students significantly and negatively predict their anxiety levels. This finding reveals the prospective protective role of self-esteem in the development of anxiety among first-generation college students, consistent with previous research (Cao and Liu, 2024), and supports the view that self-esteem serves as an important psychological resource for individuals (Song et al., 2024). Self-esteem is associated with levels of subsequent anxiety; first-generation college students with high self-esteem may possess stronger academic adaptability (Ni et al., 2025) and self-efficacy (Luo et al., 2022), enabling them to regulate anxiety more effectively when facing academic pressures and life challenges. Second, academic performance among first-generation college students significantly and negatively predicts their anxiety levels. Compared to non-first-generation college students, first-generation students tend to perform less well academically (López et al., 2023) and face greater academic pressure (House et al., 2020) and expectations (Spiegler & Bednarek, 2013). At the same time, however, they view a college education as a crucial pathway to transforming their own and their families’ futures (Tian & Zhang, 2025) and generally possess strong academic motivation and a spirit of perseverance (Yu & Han, 2018). Academic success serves as one of the sources through which they achieve self-identity and enhance their self-efficacy (Honicke et al., 2023). Good academic performance is associated with lower levels of subsequent anxiety—particularly anxiety stemming from concerns about failing to meet family expectations—which helps enhance their sense of control over the future. Conversely, academic setbacks may be associated with higher levels of subsequent anxiety, exacerbating their self-doubt. Self-esteem (β = −0.098) and academic performance (β = −0.067) have relatively small predictive effects on anxiety. However, given the large number of first-generation college students in Chinese higher education, even small effects may be significant at the population level. This suggests that anxiety among first-generation college students is influenced by a combination of multiple factors. In addition to the negative prospective associations between self-esteem, academic performance, and subsequent anxiety, omitted variables such as family socioeconomic status, accumulated cultural capital, and stable personality traits may act as co-driving factors, simultaneously influencing individual anxiety. Furthermore, research has found that anxiety does not significantly predict self-esteem or academic performance. This may be because first-generation college students have developed strong psychological resilience and stress tolerance during their upbringing (Azmitia et al., 2018). At the same time, they enter college with a strong motivation to fulfill their families’ expectations, generally demonstrating high academic motivation and a strong work ethic (Yu & Han, 2018), which to some extent weakens the predictive effect of anxiety on self-esteem and academic performance. It should be noted that academic performance in this study was measured using self-reported class rank categories, which are a relatively coarse indicator. Although this measure is widely used in Chinese educational research and shows a statistically significant prospective association with anxiety, this small effect size should be interpreted with caution. More refined or objective measures of academic performance, such as GPA or standardized test scores, might reveal different patterns of association. Therefore, our findings primarily suggest a weak but reliable prospective link rather than a strong predictive relationship.

First-generation college students are beneficiaries of the expansion of higher education and a vital force driving social development. This study provides an empirical theoretical basis for in-depth exploration of the longitudinal predictors of anxiety among this group and for promoting their comprehensive development. It also offers a new perspective on student development and educational support in higher education institutions, holding significant practical implications. First, it is recommended that universities establish student growth portfolios. By dynamically tracking changes in students’ psychological well-being and academic performance, universities and faculty can focus on first-generation college students with low self-esteem and poor academic performance, thereby identifying potential anxiety risks in a timely manner. Second, targeted growth support should be provided to first-generation college students by addressing both self-esteem and academic support. On the one hand, universities can establish diverse development platforms to encourage first-generation college students to actively participate in volunteer work and social practice, thereby enhancing their sense of accomplishment and self-esteem. On the other hand, by optimizing academic support systems—such as strengthening academic tutoring and establishing peer support mechanisms—institutions can enhance first-generation college students’ sense of academic efficacy and adaptability, thereby helping identify and support students who may be at risk of anxiety associated with academic setbacks. In addition, given that parents of first-generation college students generally lack knowledge about higher education and struggle to provide effective support, universities can establish dedicated communication channels between home and school to foster a collaborative educational effort, thereby helping identify anxiety-related risks and psychological burdens associated with family expectations at an early stage.

## 6. Limitations

This study has certain limitations. First, the representativeness of the sample needs to be further expanded; future research should consider using more representative cross-cultural, multi-center, and diverse samples to enhance the external validity of the findings. Second, regarding measurement tools, the scales used in this study are all self-report questionnaires, and the measurement of academic performance also relies on students’ self-reports. Although these scales have good reliability and validity and are widely recognized, and relative class rank is a commonly used indicator of academic performance, they are all susceptible to recall bias or social desirability bias, posing a risk of common method variance. Furthermore, in this study, although the academic performance variable was treated as a continuous observed variable, it is in fact an ordinal proxy measure; moreover, relative class rank may not fully reflect students’ underlying abilities, learning engagement, or performance in specific subject areas. Future research could incorporate teacher evaluations, standardized test scores, or more detailed learning behavior data to obtain a more comprehensive and objective assessment of academic performance. Third, this study employs a traditional two-wave cross-lagged model. While this approach helps explore predictive relationships among variables, it cannot fully distinguish between stable differences between individuals and dynamic changes within individuals, nor can it establish causal inferences. Fourth, because formal longitudinal measurement invariance testing was not conducted, observed mean-level differences across T1 and T2 were interpreted descriptively and cautiously rather than as definitive evidence of latent mean change. Fifth, although the participants in this study came from multiple universities, they likely formed clusters within their respective institutions or classes. The current analysis did not adjust standard errors for clustering, which may affect the precision of the estimates. Finally, the study did not include moderating variables that may simultaneously influence self-esteem, academic performance, and anxiety—such as personality traits, social support, and family socioeconomic status—and thus cannot completely rule out the potential for omitted-variable bias. Future research could adopt a design involving three or more waves of intensive follow-up, incorporate additional potential confounders and moderators, construct a more comprehensive theoretical model, and more accurately elucidate the longitudinal predictive relationships and underlying mechanisms among these variables.

## 7. Conclusions

Through a longitudinal survey of first-generation college students in China, this study systematically examined the prospective associations between self-esteem, academic performance, and subsequent anxiety. First, compared to T1, first-generation college students’ self-esteem levels declined slightly at T2, while their academic performance improved slightly; their anxiety levels remained within the mild range and showed a trend of gradual alleviation. Second, there was a significant negative correlation among self-esteem, academic performance, and anxiety among first-generation college students. Third, higher self-esteem and better academic performance were prospectively associated with lower subsequent anxiety among first-generation college students. These findings provide longitudinal evidence for understanding anxiety among first-generation college students, indicating a prospective association among self-esteem, academic performance, and anxiety. Higher education institutions should incorporate these two measures into comprehensive mental health intervention systems, focusing particularly on first-generation college students with low self-esteem and academic difficulties. Through targeted psychological counseling and academic support, these institutions can help foster their mental health development.

## Figures and Tables

**Table 1:** Descriptive statistics and correlations of Self-Esteem, Academic Performance, and Anxiety.

Variables	Mean	Standard Deviation	1	2	3	4	5	**6**
1. Self-esteem (T1)	36.377	6.144	1					
2. Academic Performance (T1)	6.334	2.41	0.185 *	1				
3. Anxiety (T1)	8.192	5.999	−0.412 *	−0.073 *	1			
4. Self-esteem (T2)	36.271	5.912	0.570 *	0.133 *	−0.289 *	1		
5. Academic Performance (T2)	6.554	2.357	0.157 *	0.824 *	−0.064 *	0.154 *	1	
6. Anxiety (T2)	7.828	6.129	−0.327 *	−0.129 *	0.502 *	−0.436 *	−0.130 *	1

Note: * 5% significance level.

**Table 2:** Fit indices of the models.

Model	χ^2^	DF	RMSEA (90% CI)	SRMR	CFI	TLI	Comparison	Δχ^2^	*p*
Model 1	447.09	70	0.072 [0.066–0.078]	0.058	0.954	0.941			
Model 2	429.513	68	0.071 [0.065–0.078]	0.047	0.956	0.942	M1-M2	17.577	<0.05
Model 3	442.513	68	0.073 [0.066–0.079]	0.054	0.955	0.939	M1-M3	4.577	>0.05
Model 4	426.736	66	0.072 [0.066–0.079]	0.045	0.956	0.940	M1-M4	20.354	<0.05

Note: (1) RMSEA root mean square error of approximation, SRMR standardized root mean square residual, CFI comparative fit index, TLI Tucker–Lewis index; 90% CI 90% confidence intervals. (2) χ^2^ is the robust estimate obtained using MLR. χ^2^ is strongly influenced by the sample size, so the value of χ^2^/df is not informative in this study because of the large sample size.

**Table 3:** Overview of the standardized autoregressive and cross-lagged coefficients.

Model	Autoregressive Path	β	Cross-Lagged Path	β
Model 1	Self-esteem (T1) → Self-esteem (T2)	0.560 *		
	Academic Performance (T1) → Academic Performance (T2)	0.827 *		
	Anxiety (T1) → Anxiety (T2)	0.574 *		
Model 2	Self-esteem (T1) → Self-esteem (T2)	0.579 *	Self-esteem (T1) → Anxiety (T2)	−0.106 *
	Academic Performance (T1) → Academic Performance (T2)	0.828 *	Academic Performance (T1) → Anxiety (T2)	−0.067 *
	Anxiety (T1) → Anxiety (T2)	0.518 *		
Model 3	Self-esteem (T1) → Self-esteem (T2)	0.525 *	Anxiety (T1) → Self-esteem (T2)	−0.075 *
	Academic Performance (T1) → Academic Performance (T2)	0.827 *	Anxiety (T1) → Academic Performance (T2)	−0.003
	Anxiety (T1) → Anxiety (T2)	0.586 *		
Model 4	Self-esteem (T1) → Self-esteem (T2)	0.551 *	Self-esteem (T1) → Anxiety (T2)	−0.098 *
	Academic Performance (T1) → Academic Performance (T2)	0.828 *	Academic Performance (T1) → Anxiety (T2)	−0.067 *
	Anxiety (T1) → Anxiety (T2)	0.532 *	Anxiety (T1) → Self-esteem (T2)	−0.058
			Anxiety (T1) → Academic Performance (T2)	−0.003

Note: * 5% significance level.

## Data Availability

Data will be made available upon reasonable request.
